# Variations of circulating cardiac biomarkers during and after anthracycline-containing chemotherapy in breast cancer patients

**DOI:** 10.1186/s12885-018-4015-4

**Published:** 2018-01-29

**Authors:** Pierre Frères, Nassim Bouznad, Laurence Servais, Claire Josse, Stéphane Wenric, Aurélie Poncin, Jérôme Thiry, Marie Moonen, Cécile Oury, Patrizio Lancellotti, Vincent Bours, Guy Jerusalem

**Affiliations:** 1 Department of Medical Oncology, University Hospital (CHU) and University of Liège, Liège, Belgium; 2 Laboratory of Human Genetics, GIGA Research, University Hospital (CHU) and University of Liège, Liège, Belgium; 3 GIGA Cardiovascular Sciences, Department of Cardiology, Heart Valve Clinic, University Hospital (CHU) and University of Liège, Liège, Belgium; 4 Gruppo Villa Maria Care and Research, Anthea Hospital, Bari, Italy

**Keywords:** Biomarkers, Cardiotoxicity, Chemotherapy, Soluble ST2, microRNAs

## Abstract

**Background:**

Over time, the chance of cure after the diagnosis of breast cancer has been increasing, as a consequence of earlier diagnosis, improved diagnostic procedures and more effective treatment options. However, oncologists are concerned by the risk of long term treatment side effects, including congestive heart failure (CHF).

**Methods:**

In this study, we evaluated innovative circulating cardiac biomarkers during and after anthracycline-based neoadjuvant chemotherapy (NAC) in breast cancer patients. Levels of cardiac-specific troponins T (cTnT), N-terminal natriuretic peptides (NT-proBNP), soluble ST2 (sST2) and 10 circulating microRNAs (miRNAs) were measured.

**Results:**

Under chemotherapy, we observed an elevation of cTnT and NT-proBNP levels, but also the upregulation of sST2 and of 4 CHF-related miRNAs (miR-126-3p, miR-199a-3p, miR-423-5p, miR-34a-5p). The elevations of cTnT, NT-proBNP, sST2 and CHF-related miRNAs were poorly correlated, suggesting that these molecules could provide different information.

**Conclusions:**

Circulating miRNA and sST2 are potential biomarkers of the chemotherapy-related cardiac dysfunction (CRCD). Nevertheless, further studies and long-term follow-up are needed in order to evaluate if these new markers may help to predict CRCD and to identify the patients at risk to later develop CHF.

**Electronic supplementary material:**

The online version of this article (10.1186/s12885-018-4015-4) contains supplementary material, which is available to authorized users.

## Background

The cancer burden is a worldwide major public health problem. Fortunately, the outcome, including the cancer death rate, can be improved by earlier diagnosis and better treatment [1].

Because more patients are cured, the attention is now focusing on quality of life and long-term outcome of cancer survivors. Cardiovascular disease is the leading cause of late mortality among survivors of childhood and adolescent cancer. The risk of cardiovascular death is higher than the actual risk of cancer recurrence in many adult cancer patients in complete remission. Cancer survivors have a ten-fold higher mortality than the general population, with a fifteen-fold increased risk of developing a congestive heart failure (CHF) and a ten-fold increased risk of coronary artery disease [2, 3]. These patients also have a higher risk of atherosclerosis, hypertension, pericardial disease, valvular heart disease and dyslipidemia [4, 5]. The higher cardiovascular death rate in cancer survivors is secondary to a combination of cancer treatment-related risk effects (ionizing irradiation, cytotoxic and targeted agents), familiar risk factors and health behavior. Consequently, cancer survivors need appropriate surveillance in order to early detect long-term side effects of cancer therapy, allowing appropriate treatment before the toxicity becomes irreversible [4, 5].

In this respect, easily accessible circulating biomarkers could be seen as highly valuable diagnostic tools for early detection of cardiotoxicity related to cancer treatments.

The cardiac-specific isoenzymes of troponins T and I (cTnT, cTnI) are released into the blood when cardiomyocytes are damaged. Troponin levels rapidly increase after chemotherapy and might predict late cardiac events [6]. However, troponins release is related to lysis of myocardial cells, which may be initially absent in case of type II heart damage chemotherapy. In addition, their half-life is relatively short (2 h) [7], requiring the collection of multiple blood samples during the treatment, which is hardly feasible during outpatient’s treatments.

N-terminal brain natriuretic peptides (NT-proBNP) are released by left ventricular cardiomyocytes in response to wall stress. This biomarker has well-established clinical utility in CHF. NT-proBNP elevation during chemotherapy in breast cancer patients has been related to asymptomatic decline in left ventricular ejection fraction (LVEF) [8–11]. Nevertheless, studies evaluating the predictive role of NT-proBNP in the detection of chemotherapy-related cardiac dysfunction (CRCD) gave conflicting results and the threshold of positive tests has not been determined yet [12, 13].

Soluble ST2 (sST2), a member of the interleukin-1 receptor family, is a more recently discovered biomarker of cardiovascular stress. Two studies have demonstrated that high levels of sST2 may be a strong predictor of cardiovascular death in CHF patients [14, 15]. Furthermore, sST2 has a higher discrimination power than NT-proBNP in CHF patients [16], and in community-based populations free of cardiovascular disease [17].

MicroRNAs (miRNAs) are approximately 22-nucleotide long RNAs that regulate gene expression by binding to and consequently silencing target messenger RNAs. They are involved in multiple biological processes including cell proliferation, differentiation and apoptosis. All cell types release miRNAs in peripheral blood under both normal and pathological conditions. Therefore, circulating miRNAs are promising biomarkers for the early and minimally invasive diagnosis of cancer and its treatment-related cellular toxicity [18–24].

The use of anthracyclines in the treatment of breast cancer is limited by dose-dependent cardiotoxicity, which eventually may lead to CHF. The aim of the present study is to evaluate the variations of innovative biomarkers during and after anthracycline-containing chemotherapy. Plasma levels of cTnT, NT-proBNP, sST2 and 10 selected miRNAs were measured in a total of 45 breast cancer patients receiving anthracyclines as part of their neoadjuvant chemotherapy (NAC). The neoadjuvant setting was selected because patients were treatment-naïve, so that markers levels were not modified by other therapies such as surgery or radiotherapy.

## Methods

### Population

Ethics approval was obtained from the local Institutional Review Board and the Ethic Committee (Ethical Committee of the Faculty of Medicine of the University of Liège). This prospective study was performed in compliance with the Declaration of Helsinki. Patients with treatment-naive primary breast cancer (*n* = 45, median age = 49 years, range = 26–78 years) were recruited prospectively at CHU of Liège (Liège, Belgium) from 7/2011 to 9/2014. All patients signed a written informed consent form. Biomarker results were not communicated to the treating physicians and consequently did not lead to any change in treatment. Patients and tumor characteristics are summarized in Table 1.

**Table 1:** Characteristics of patients and tumors (NA = not assessed, IDC = invasive ductal carcinoma, ILC = invasive lobular carcinoma)

Characteristics	Primary breast cancer patients (*n* = 45)
Median age (range) (y)	49 (26–78)
Estrogen receptor [n (%)]	29 (64)
Progesterone receptor [n (%)]	26 (58)
HER2 [n (%)]	17 (38)
Ki67 (median ± SD) (%)	30 ± 24
Initial T staging [n (%)]	
NA	0
1	4 (9)
2	23 (51)
3	7 (16)
4	11 (24)
Lymph node involvement [n (%)]	33 (73)
Tumor node metastasis (TNM) stage [n (%)]	
NA	0
1	0
2	26 (58)
3	19 (42)
Scarff-Bloom-Richardson grading system [n (%)]	
NA	0
1	0
2	19 (42)
3	26 (58)
Histologic subtype [n (%)]	
NA	0
IDC	42 (93)
ILC	3 (7)
Others	0
Lymphovascular invasion [n (%)]	9 (20)
Cardiovascular risk factors [n (%)]	
Smoking	12 (27)
Type 2 diabetes	4 (9)
High blood pressure	9 (20)
Obesity	7 (16)
Dyslipidemia	21 (47)
Chronic kidney disease	2 (4)
Left ventricular ejection fraction (median ± SD) (%)	
Before chemotherapy	64 ± 11
After chemotherapy	60 ± 29

### Chemotherapy treatment

All patients received NAC consisting in the sequential use of 4 courses of alkylating (cyclophosphamide) and anthracycline-based (epirubicin) chemotherapy followed by 9 to 12 weeks of tubulin-binding agent (paclitaxel) based chemotherapy. Seventeen patients suffering from HER2 amplified breast cancer received, in addition, targeted therapy (trastuzumab or lapatinib) administered concomitantly with tubulin-binding agents.

### Plasma collection

**Fig. 1:**
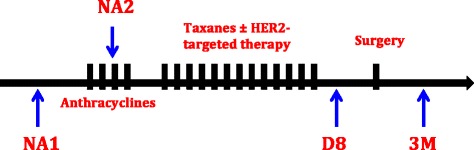
Blood samples were drawn at baseline before neoadjuvant chemotherapy (NA1), after 2 cycles of anthracycline-containing chemotherapy (NA2), at the end of the chemotherapy 8 days before surgery (D8) and 3 months after the surgery (3 M)

Blood samples were drawn at baseline before NAC (NA1), after 2 cycles of anthracycline-containing chemotherapy (NA2), at the end of the chemotherapy 8 days before surgery (D8) and 3 months after the surgery (3 M), as shown in Fig. 1. Plasma was collected in 9 ml EDTA tubes, was prepared within 1 h by retaining supernatant after double centrifugation at 4 °C (10 min at 815 g and 10 min at 2500 g) and was stored at − 80 °C.

### Plasma concentration of cardiac-specific troponins T, N-terminal brain natriuretic peptides and soluble ST2

cTnT and NT-proBNP were assessed in plasma with highly sensitive third-generation quantitative test (electrochemoluminescence method ECLIA, Roche Diagnostics, Belgium), as recommended by the manufacturer. Detection limit was 14 μg/L for cTnT and 400 ng/mL for NT-proBNP.

The concentrations of sST2 in the plasma were measured using a IL-1 R4/ST2 enzyme-linked immunosorbent assay (R&D Systems, United Kingdom), with a mean minimal detectable dose of 5.1 pg/ml. The inter- and intra-assay variation was 6% and 5%, respectively.

### Selection, extraction and qRT-PCR of microRNAs

Based on previous publications, potentially interesting miRNAs in the context of the CRCD were selected. Three groups of miRNAs were determined: *i)* Acute myocardial infarction (AMI)-related miRNAs, including miR-1, miR-133a, miR-133b and miR-499-5p [25]; *ii)* CHF-related miRNAs, including miR-208a, miR-208b, miR-126-3p, miR-199a-3p and miR-423-5p [26–28]; and *iii)* miR-34a-5p, which is highly upregulated after anthracycline treatment [23] and correlated with cardiac aging and function [29].

Essential MIQE (Minimum Information for Publication of Quantitative Real-Time PCR Experiments) guidelines were followed during specimen preparation [30].

Circulating miRNAs were purified from 100 μl of plasma with the miRNeasy mini kit (Qiagen, Germany) according to the manufacturer’s instructions. The standard protocol was adapted on the basis of Kroh’s recommendations [31]. MS2 (Roche, Belgium) was added to the samples as a carrier, cel-miR-39 and cel-miR-238 were added as spike-ins. RNA was eluted in 50 μl of RNase-free water at the end of the procedure.

Reverse transcription was performed using the miRCURY LNA™ Universal RT microRNA PCR, polyadenylation and cDNA synthesis kit (Exiqon, Denmark). Quantitative PCR was performed according to the manufacturer’s instructions on custom panels with the 10 selected miRNAs (Pick-&-Mix microRNA PCR Panels, Exiqon). Controls included the reference genes described in the text, inter-plate calibrators in triplicate (Sp3) and negative controls.

All PCR reactions were performed on a LightCycler 480 Real-Time PCR System (Roche, Belgium). miRNAs were considered for analysis with a quantification cycle (Cq) value < 36.

### Data analysis

Analyses were conducted using the 2^-ΔCq^ method (ΔCq = Cq_sample_ – Cq_reference gene_) for each sample to obtain a normalized expression value of the miRNAs [32]. Data were then normalized using the ΔCq method as recommended by Mestdagh et al. [33]. The mean Cq of the 3 most stable miRNAs across NAC-treated patients (miR-484, miR-652 and miR-148b according GeNorm analysis of previous published data) [23] was used as reference genes in addition to the cel-miR-39 spike-in.

Statistical analyses were performed with the GraphPad Prism software, version 6.00 (GraphPad Software, USA) (www.graphpad.com/scientific-software/prism/). The normal distribution of values was evaluated with the D’Agostino-Pearson omnibus and Shapiro-Wilk tests. To compare marker levels, Student’s t-test (two-tailed) and non-parametric two-sided Wilcoxon or Mann-Whitney *U* tests were used. Correlations between continuous variables were assessed with the Spearman test. Statistical significance was established as *p* < 0.05 (*), *p* < 0.01 (**), *p* < 0.001 (***) or *p* < 0.0001 (****).

## Results

### Neoadjuvant chemotherapy induces high plasma level expression of cardiac-specific troponins T, N-terminal brain natriuretic peptides and soluble ST2

**Fig. 2:**
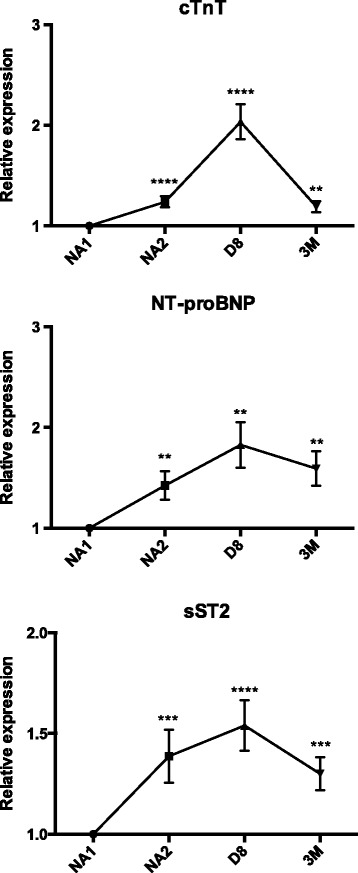
Cardiac-specific troponins T (cTnT), N-terminal natriuretic brain peptides (NT-proBNP) and soluble ST2 (sST2) relative levels (mean fold change) during the neoadjuvant chemotherapy in 45 breast cancer patients. Comparisons between the initial and subsequent time points were calculated using the Wilcoxon tests

cTnT were initially undetectable in most patients, with a median level under the detection limit at baseline (NA1 time point). Their levels were then increased with a 1.3-fold at NA2 (*p* < 0.0001), a 2-fold at D8 (*p* < 0.0001) and a 1.2-fold at 3 M (*p* < 0.01). An elevation in cTnT levels was demonstrated in 42% of patients at NA2, 73% at D8 and 47% at 3 M (Fig. 2, Table 2).

**Table 2:** Percentage of significant increase in markers levels at each time point of the chemotherapy treatment

Time points	cTnT	NT-proBNP	sST2	miR-126-3p	miR-199a-3p	miR-423-5p	miR-34a-5p
NA2	42%	58%	64%	76%	64%	78%	98%
D8	73%	60%	87%	–	–	78%	89%
3 M	47%	60%	69%	71%	–	73%	89%

NT-proBNP were found to be 1.4-fold elevated at NA2, 1.8-fold elevated at D8 and 1.6-fold elevated at 3 M (*p* < 0.01 for each time point). The concentrations of NT-proBNP were increased in 58% of patients at NA2 and 60% of patients at D8 and 3 M (Fig. 2, Table 2).

sST2 levels were increased by 1.4-fold in 64% of patients at NA2 (*p* < 0.001), by 1.6-fold in 87% of patients at D8 (*p* < 0.0001) and by 1.3-fold in 69% of patients at 3 M (*p* < 0.001) (Fig. 2, Table 2).

### Cardiac heart failure-related microRNAs plasma levels significantly increase after neoadjuvant chemotherapy

None of the AMI-related miRNAs (miR-1, miR-133a, miR-133b, miR-499-5p) were significantly deregulated during and after the NAC.

**Fig. 3:**
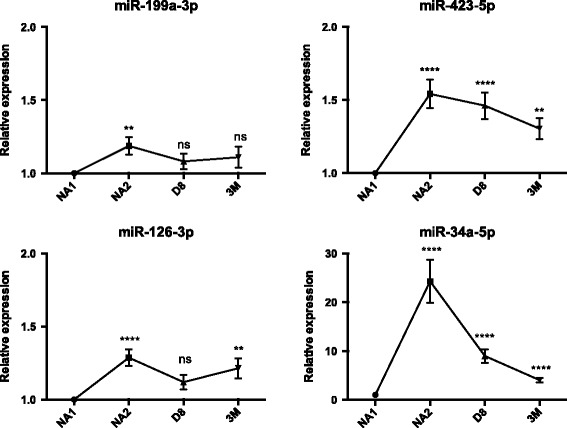
The relative level of microRNAs (mean fold change) during the neoadjuvant chemotherapy (NAC) in breast cancer patients. Plasma levels of microRNAs were determined by RT-qPCR in the plasma of 45 NAC-treated patients. Comparisons between the initial and subsequent time points were calculated using the Wilcoxon tests

Significant increases were found regarding the levels of CHF-related miRNAs. The concentrations of miR-126-3p were elevated by 1.3-fold in 76% of patients at NA2 (*p* < 0.0001) and in 71% of patients at 3 M. This miRNA was not significantly deregulated at D8 (*p* > 0.05). miR-199a-3p levels were found elevated by 1.2-fold in 64% of patients at NA2 (*p* < 0.01) but were not modified at D8 and 3 M time points. miR-423-5p levels were increased by 1.6-fold in 78% of patients at NA2 (*p* < 0.0001), by 1.5-fold in 78% of patients at D8 (*p* < 0.0001) and by 1.3-fold in 73% of patients at 3 M (*p* < 0.01). On the other side, miR-208a and miR-208b levels were not deregulated during the NAC (Fig. 3, Table 2).

Finally, miR-34a-5p was confirmed to be sensitive to anthracycline-based chemotherapy with a 24.3-fold increase at NA2 in 98% of patients. miR-34a-5p levels were also increased by 9-fold at D8 and 4.1-fold at 3 M, in 89% of patients. The increase of miR-34a-5p was highly significant at each time point (*p* < 0.0001) (Fig. 3, Table 2).

### Correlations between cardiac biomarkers and miRNAs

Relationships between changes in the plasma levels of the biomarkers were evaluated (see Additional file 1 for the statistical analyses). The elevation of cTnT was significantly correlated with that of NT-proBNP (*p* < 0.01, *r* = 0.46) and sST2 (*p* < 0.001, *r* = 0.48) at D8. No correlation was found between the elevation of NT-proBNP and sST2. The only correlation observed between miRNAs and the other biomarkers concerns the increase of cTnT and miR-199a-3p at D8 (*p* < 0.05, *r* = 0.31), and the rise of sST2 that is inversely correlated with that of miR-423-5p at 3 M (*p* < 0.05, *r* = − 0.29).

### Correlations between biomarkers variations and clinical data

All patients had normal LVEF before starting chemotherapy. A significant decline in LVEF, defined as ≥10% decline from baseline to ≤55% [34], has been noted in 7 patients (16% of the cohort), on average 20 months after the initial ultrasound. Of these 7 patients, 6 had HER2-positive breast cancer and degraded their LVEF during or after anti-HER2 treatment. One of these patients finally developed a CHF, with an LVEF evaluated at 33%, while she was still under adjuvant anti-HER2 treatment.

**Fig. 4:**
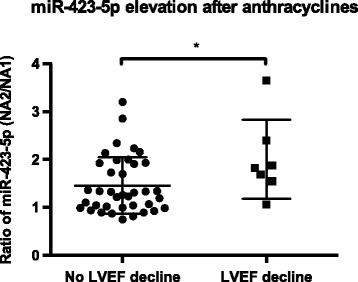
Comparison of circulating miR-423-5p elevation directly after anthracyclines between patients with (*n* = 7) or without (*n* = 38) LVEF decline. Expression was determined by RT-qPCR at NA1 and NA2 time points. Comparisons between the 2 groups were calculated using the Mann-Whitney *U* test. The data are expressed as the mean ± SEM

Biomarkers variations were compared between the 2 groups of patients (normal vs. decreased LVEF). No significant differences for cTnT, NT-proBNP and sST2 were found. However, the patient who developed a CHF had higher-than-average values for these 3 markers, particularly at the end of chemotherapy for sST2 (D-8 time point, 3.64 vs. 1.53 pg/mL, SD = 0.84) and 3 month after surgery for NT-proBNP (3 M time point, 6.54 vs. 1.55 ng/mL, SD = 1.14). For miRNAs, the elevation of miR-423-5p directly after anthracyclines (NA2 time point) was significantly greater in patients with decreased LVEF (*p* = 0.045, 1.28-fold, Fig. 4). The patient who developed a CRCD also had a higher elevation of miR-423-5p than the mean of other patients (2.39 vs. 1.54-fold, SD = 0.65).

### HER2-targeted therapies do not modify cardiac biomarkers plasma levels

In our cohort, 17 patients with HER2-positive breast cancer received HER2-targeted therapy during the neoadjuvant setting (trastuzumab or lapatinib). HER2-targeted therapies are associated with a modest risk of reversible cardiotoxicity, which is typically observed as an asymptomatic decrease in left ventricular function [35]. We did not observe any difference in biomarker modifications in these patients as compared with HER2-negative patients (see Additional file 1).

## Discussion

cTnT and NT-proBNP are both important biomarkers in heart diseases. Our study finds a significant increase in these biomarkers at the end of the NAC for breast cancer, and potentially identifies a group of patients at risk of CRCD.

Compared with cTnT and NT-proBNP, sST2 levels increase in a higher percentage of patients directly after anthracyclines-based chemotherapy (NA2 time point). As sST2 is strongly associated with CHF severity and outcome [14, 15], its blood concentration during chemotherapy may be an important predictor of long-term CRCD. Nonetheless, sST2 lacks tissue specificity and its levels could be elevated in case of breast cancer [36, 37]. In fact, Lu et al. previously reported elevated sST2 levels in the serum of breast cancer patients, with a decrease after tumor surgery [36]. All our patients experienced a complete - or at least a partial - pathologic response to the NAC, and were free of disease after 3 months. If tumor cells indeed secreted plasma sST2, we would expect a progressive decrease in its levels as the tumor responds or after the surgery. On the contrary, plasma levels of sST2 remain significantly higher, compared with baseline, after the chemotherapy and even 3 months after breast surgery (D8 and 3 M time points, Fig. 2). Apoptotic cancer cells could also have released plasma sST2. If this was the case, we should detect a correlation between sST2 upregulation and tumor response to chemotherapy, but such a correlation was not found. Based on these results, we think that sST2 elevation is a consequence of the chemotherapy rather than a reflection of the tumor presence and/or response.

Among selected miRNAs, we demonstrated the increase of 3 miRNAs related to the diagnosis and prognosis of CHF: miR-126-3p, miR-199a-3p, miR-423-5p. Vascular endothelium-enriched miR-126 has been associated with coronary artery disease and CHF. Reduced levels were observed in the acute phase [26, 28, 38], followed by normalization after clinical improvement [26, 28]. CRCD does not necessarily imply tissue ischemia [18] and an increase, rather than a decrease, of miR-126-3p concentrations was found after the chemotherapy. As miR-126-3p is enriched in endothelial cells and promotes blood vessels formation [39], its upregulation could be a response to cellular stress and anti-angiogenic activity of the chemotherapy [40]. In the same way, miR-199a-3p levels also decreased in acute heart failure [41], while our results have shown an elevation after chemotherapy. miR-199a-3p is largely expressed in cardiomyocytes and its myocardial levels are upregulated in hypertrophic hearts [42], which is sometimes observed in CRCD [18]. After myocardial infarction in mice, miR-199a promotes cardiomyocytes regeneration and recovery of cardiac functional parameters [43]. Cardiomyocytes could therefore release miR-199a-3p under the effect of the chemotherapy to play a cardio-protective role, though this hypothesis requires further explorations. Plasma levels of miR-423-5p were highly increased immediately after anthracyclines (NA2 time point), especially in patients with decreased LVEF (Fig. 4). Two recent studies have unveiled that miR-423-5p was enriched in the blood of patients suffering from CHF, with a high diagnosis power and a significant correlation with CHF prognostic markers [27, 44]. In another study, circulating miR-423-5p levels were able to predict long-term mortality of CHF patients [45]. Based on these reports and our findings, we believe that the diagnostic, prognostic and predictive roles of miR-423-5p in CRCD should be further explored.

Circulating miR-34a-5p was also studied for multiple reasons. Boon et al. demonstrated that miR-34a was involved in the alteration of cardiac contractile function after AMI, by inducing telomere attrition [29]. Desai et al. evaluated miR-34a expression in myocardial tissues of mouse exposed to increasing doses of doxorubicin. Unlike troponins, upregulation of miR-34a was an early event inside the cardiac tissue and did not involve prior necrosis of cardiomyocytes [20]. Our study indicates a strong increase in miR-34a plasma levels immediately after anthracycline-based chemotherapy, followed by a gradual decrease. The tumor suppressor p53 is known to promote both growth arrest and apoptosis upon DNA damage [46]. miR-34a is directly induced by p53 to exert anti-tumor functions [47]. This might explain the miR-34a upregulation observed after anthracyclines, which cause DNA breaks and p53 activation [48]. The subsequent tubulin-binding agent based chemotherapy does not act via DNA breaks but by the disruption of microtubule function. Plasma miR-34a increase could therefore be specific of anthracyclines chemotherapy, explaining the gradual decrease of miR-34a levels after this part of the treatment. The concentration of miR-34a-5p may help for the identification of patients at risk of developing CRCD, however, the threshold of a positive test is still unknown. One problem may be the significant elevation in a high percentage of patients although most of them will not develop any cardiotoxicity. Furthermore, miR-34a is broadly expressed in normal tissues and this miRNA may thus lack specificity. The increase of miR-34a levels after anthracyclines was not correlated with the increase of troponins. This observation is in contradiction with our previous study that was performed on a smaller number of patients [23], probably because of the limited statistical power. Globally, the elevation of the other markers (cTnT, NT-proBNP, sST2) was poorly correlated with that of CHF-related miRNAs, which could imply that these molecules provide distinct information about CRCD.

Interpretation of our results is limited for several reasons. Firstly, the small number of patients, with only one who developed a clinical CRCD, does not allow definitive conclusions to be drawn. In addition, majority of patients experienced an asymptomatic decline in LVEF in the context of anti-HER2 therapy, and we know that the cardiotoxicity mechanism is different from that of anthracyclines [18]. As cardiomyopathy is a late side effect of the chemotherapy, a long-term follow-up is required. The biomarkers we identified (sST2, miR-126-3p, miR-199a-3p, miR-423-5p and miR-34a-5p) should therefore be studied in a larger prospective trial with regular and prolonged cardiac monitoring. These biomarkers may be useful either as a predictive marker of cardiotoxicity at the time of treatment or as a new tool for the early identification of late side effects before the patients have clinical symptoms. Importantly, appropriate treatment of CRCD may prevent irreversible consequences if initiated early.

## Conclusion

We identified sST2, miR-126-3p, miR-199a-3p, miR-423-5p and miR-34a-5p as innovative biomarkers for potential early and sensitive detection of the cardiomyopathy associated with anthracycline-based breast cancer chemotherapy.

## Additional file

Additional file 1: Statistical analyses. (A) Correlations Spearman tests between the fold change of cTnT, NT-proBNP, sST2, miR-126-3p, miR-199a-3p, miR-423-5p and miR-34a-5p at different time point of the neoadjuvant chemotherapy in 45 breast cancer patients. (B) Comparison between the increase of the markers at the end of the chemotherapy (NA1 vs. D8 and NA1 vs. 3 M) in patients treated (*n* = 17) or not (*n* = 28) with HER2 targeted therapy, using Mann-Whitney tests. (XLSX 50 kb)

## Abbreviations

AMI: Acute myocardial infarction
CHF: Congestive heart failure
CRCD: Chemotherapy-related cardiac dysfunction
cTnT: Cardiac-specific troponins T
LVEF: Left Ventricular Ejection Fraction
MIQUE: Minimum Information for Publication of Quantitative Real-Time PCR Experiments
miRNAs: microRNAs
NAC: Neoadjuvant chemotherapy
NT-proBNP: N-terminal brain natriuretic peptides
SD: Standard deviation
SEM: Standard error of the mean
sST2: Soluble ST2
